# Regulation of vascular growth and tone by hydrogen sulfide

**DOI:** 10.1186/1471-2210-11-S1-O27

**Published:** 2011-08-01

**Authors:** Andreas Papapetropoulos

**Affiliations:** 1 Laboratory for Molecular Pharmacology, Department of Pharmacy, University of Patras, Patras 26504 Greece

## 

Hydrogen sulfide (H_2_S) is a colourless, flammable gas with a characteristic pungent smell. Until recently, the H_2_S literature was focused in the toxicology of this agent. Exposure to H_2_S concentrations lower than 100 ppm lead to eye irritation, sore throat, dizziness, nausea, shortness of breath, and chest tightness, while exposure to 1000 ppm or more causes central nervous system toxicity and respiratory depression. In the past 5 years the interest for H_2_S biology has seen an upsurge, as it was accepted that low amounts of this gas are endogenously produced in most mammalian tissues. Increasing evidence shows that H_2_S acts as a signalling molecule in cells and is now considered the third member of the gasotransmitter family along with nitric oxide and carbon monoxide. Most of H_2_S is produced by two enzymes cystathionine-synthase (CBS) and cystathionine γ-lyase (CSE) that use L-cysteine as a substrate and pyridoxal phosphate as a co-factor. CBS is highly expressed in the central nervous system, while CSE is abundantly present in the heart, lung, blood vessels, liver and kidney.

In the cardiovascular system, H_2_S has anti-apoptotic effects on cardiomyocytes, exerts cardioprotective actions and modifies vascular tone. We have recently shown that exogenously administered Na_2_S stimulates endothelial proliferation, migration and capillary-like network formation. These effects of H_2_S on migration are mediated through the activation of ATP-sensitive K^+^-channels (K_ATP_) and activation of mitogen activated protein kinase pathways. Inhibition of CSE and reduction of endogenously produced H_2_S reduces vascular network length and branching of blood vessels in the chicken chorioallantoic membrane. Exposure of endothelial cells to vascular endothelial growth factor (VEGF) increased H_2_S production and genetic or pharmacological inhibition of CSE restricted VEGF signalling and VEGF-driven angiogenic responses (migration, sprouting). Thus, H_2_S is an endogenous activator of angiogenesis and modulation of its production might be useful in diseases characterized by aberrant or excessive angiogenesis.

H_2_S exerts important biological actions in vascular smooth muscle cells. Originally H_2_S–induced vasorelaxation was believed to be mediated by potassium channels (including K_ATP_ channels). However, significant vasodilation can be observed after sulfonylurea treatment, suggesting that additional pathways are involved. We recently showed that H_2_S donors inhibit phosphodiesterase activity in vitro and that exposure to H_2_S increases cGMP levels in smooth muscle cells. The cGMP elevating effects of H_2_S are abolished following phosphodiesterase inhibition. Moreover, incubation of cells with H_2_S led to an increase in VASP phopshorylation on Ser239, suggesting that H_2_S activates PKG signalling pathways. More importantly, incubation of aortic rings with selective PKG-I inhibitors (DT-2, DT-3) attenuated H_2_S-induced vasorelaxation. Our results reinforce the notion that H_2_S plays important roles in vascular biology, by modulating vascular growth and tone.

